# Hitting two oncogenic machineries in cancer cells: cooperative effects of the multi-kinase inhibitor ponatinib and the BET bromodomain blockers JQ1 or dBET1 on human carcinoma cells

**DOI:** 10.18632/oncotarget.25474

**Published:** 2018-05-29

**Authors:** Karin Bauer, Daniela Berger, Christoph C. Zielinski, Peter Valent, Thomas W. Grunt

**Affiliations:** ^1^ Department of Medicine I, Division of Hematology and Hemostaseology, Medical University of Vienna, Vienna, Austria; ^2^ Ludwig Boltzmann Cluster Oncology, Medical University of Vienna, Vienna, Austria; ^3^ Comprehensive Cancer Center, Medical University of Vienna, Vienna, Austria; ^4^ Department of Medicine I, Division of Oncology, Medical University of Vienna, Vienna, Austria

**Keywords:** BRD4 degrader, dBET1, drug combination, JQ1, ponatinib

## Abstract

In recent years, numerous new targeted drugs, including multi-kinase inhibitors and epigenetic modulators have been developed for cancer treatment. Ponatinib blocks a variety of tyrosine kinases including ABL and fibroblast growth factor receptor (FGFR), and the BET bromodomain (BRD) antagonists JQ1 and dBET1 impede MYC oncogene expression. Both drugs have demonstrated substantial anti-cancer efficacy against several hematological malignancies. Solid tumors, on the other hand, although frequently driven by FGFR and/or MYC, are often unresponsive to these drugs. This is due, at least in part, to compensatory feedback-loops in the kinome and transcription network of these tumors, which are activated in response to drug exposure. Therefore, we hypothesized that the combination of the multi-kinase inhibitor ponatinib with transcription modulators such as JQ1 or dBET1 might overcome this therapeutic recalcitrance. Using ^3^H-thymidine uptake, cell cycle analysis, and caspase-3 or Annexin V labeling, we demonstrate that single drugs induce moderate dose-dependent growth-inhibition and/or apoptosis in colon (HCT116, HT29), breast (MCF-7, SKBR3) and ovarian (A2780, SKOV3) cancer cells. Ponatinib elicited primarily apoptosis, while JQ1 and dBET1 caused G0/G1 cell cycle arrest and very mild cell death. Phospho-FGFR and MYC, major targets of ponatinib and BET inhibitors, were downregulated after treatment with single drugs. Remarkably, ponatinib was found to sensitize cells to BET antagonists by enhancing apoptotic cell death, and this effect was associated with downregulation of MYC. In summary, our data shows that ponatinib sensitizes colon, breast, and ovarian cancer cells to BET bromodomain inhibitors. Further studies are warranted to determine the clinical value of this phenomenon.

## INTRODUCTION

The main options for treatment of solid tumors are surgery, irradiation and chemotherapy. In recent years, a much deeper understanding of the mechanisms underlying evolution and progression of various neoplasms (cancer) has been achieved. As a result, novel targeted therapies that specifically interfere with these pro-oncogenic machineries have been developed. The underlying processes and molecular pathways, summarized by Hanahan and Weinberg, are now known as the hallmarks and major drivers of cancer evolution [1, 2]. Some of these cancer drivers are targetable with newly developed drugs, which have already demonstrated significant clinical benefit, either alone or in combination [1–3]. Unfortunately, not all pro-oncogenic pathways and targets are easily accessible by specifically designed drugs [4, 5]. MYC, for example, is a well-known major driver of oncogenesis and has been identified as one of the most commonly deregulated oncogenes in a wide variety of cancer types, yet potent drugs directly targeting MYC are still not available [4, 5].

Nevertheless, a number of different epigenetic drugs directed against chromatin-regulatory molecules have recently been developed. JQ1, for instance, is a highly potent and selective inhibitor targeting bromodomain-containing proteins (BRD) of the extra-terminal domain (BET) family. This drug blocks interactions between BRD4 and acetylated histones and regulates transcription. One important effect of JQ1 is downregulation of MYC [6, 7]. In addition, small-molecule BET degraders have recently been designed, with the aim to induce rapid BET protein degradation in cancer cells. dBET1, the BET degrader used in this study, is a composite molecule consisting of JQ1, which binds to BDR4, and of a thalidomide moiety, which targets cereblon, a component of the ubiquitin ligase complex. Exposure to dBET1 results in a rapid degradation of BRD4 in target cells [8]. Although drugs against BET bromodomains such as JQ1 show remarkable antineoplastic effects in various forms of leukemia [9], their preclinical activities in advanced solid tumors are at best moderate [10]. Interestingly, the combination of MAPK- or PI3K-blockers with BRD4-MYC antagonists show sustained growth inhibition, although silencing of MAPK or PI3K only causes transient anticancer effects in most solid tumors. These results can be explained by drug interference with at least two negative feedback loops. On the one hand, BET-inhibitors can cause compensatory MAPK activation by blocking dual-specificity phosphatase (DUSP), which normally blunts MAPK activity. This effect attenuates the BET inhibitor-mediated anticancer response [11]. PI3K-targting drugs, on the other hand, suspend AKT-mediated repression of FOXO transcription factor and stimulate BRD4, which in turn can upregulate and reactivate numerous upstream kinases such as PI3K [10–12]. Therefore, one possible way to improve the anticancer response of solid tumors to kinase and BET inhibitors is to abrogate these feedback loops by interfering with oncogenic signaling upstream of MAPK and PI3K.

Ponatinib, a potent tyrosine kinase inhibitor (TKI), was originally designed to target the BCR-ABL1 oncoprotein. Subsequent work revealed that ponatinib blocks a wide range of additional oncogenic tyrosine kinases upstream of both MAPK and PI3K, including fibroblast growth factor receptor 1 (FGFR1), platelet-derived growth factor receptor (PDGFR) α, vascular endothelial growth factor receptor (VEGFR) 2, KIT, SRC, and FLT3 [13, 14]. Therefore, we reasoned that this multi-kinase inhibitor might be a most promising candidate for combination with BRD4 antagonists in solid cancer cells. So far, ponatinib has been applied for treatment of hematological malignancies such as chronic myeloid leukemia (CML) and Philadelphia chromosome-positive acute lymphoblastic leukemia (Ph+ ALL) [15]. Moreover, ponatinib has also shown some anti-neoplastic effects on various cancer cells, including endometrial, bladder, gastric, breast, lung, and colon cancer [16]. In the present study, we examined single-agent and combinatorial effects of ponatinib, JQ1 and dBET1 on proliferation, cell cycle distribution, and apoptosis in two colon, two breast and two ovarian cancer cell lines. Our data demonstrates that ponatinib significantly increases the sensitivity of carcinoma cells to BRD4 targeted drugs.

## RESULTS

### Ponatinib, JQ1 and dBET1 are moderate inhibitors of proliferation, cycle progression and survival of carcinoma cells

Ponatinib, JQ1 and dBET1 were originally developed for use in liquid tumors [8, 17, 18]. However, recent data suggests that these inhibitors may also show anti-neoplastic activity against several types of solid malignancies. We examined the effects of single treatment with ponatinib, JQ1 or dBET1 on growth of colon, mammary and ovarian cancer cell lines. Data obtained by ^3^H-thymidine uptake revealed different drug-specific anti-proliferative efficacies. While the concentrations of ponatinib causing 50% growth inhibition (IC_50_ values) were quite similar in all cell lines tested (range 0.35–1.37 µM), the IC_50_ values for JQ1 and dBET1 were different in the various cancer types (range 0.33–8.95 µM). Colon cancer cells (HCT116, HT29) were characterized by relatively high IC_50_ values for JQ1 and dBET1 (range 3.80–8.95 µM) suggesting that colon cancer is relatively resistant to BET targeting drugs, consistent with previous data [10]. Breast cancer cells (MCF7, SKBR3), on the other hand, were sensitive to JQ1 (IC_50_ range 0.33–1.10 µM), but not to dBET1 (IC_50_ range 2.19–5.90 µM), whereas ovarian cancer cells (A2780, SKOV3) were sensitive to both types of BET antagonists (IC_50_ range 0.45–1.49 µM, Table 1).

**Table 1:** Growth-inhibitory effects of ponatinib, JQ1 and dBET1 in cancer cell lines

	IC_50_ (µM)			
		Ponatinib	JQ1	dBET1
Colon cancer	HCT116	0.35 ± 0.17	6.15 ± 4.04	8.95 ± 0.67
	HT29	0.97 ± 0.02	3.80 ± 0.48	5.19 ± 4.76
Breast cancer	MCF7	1.37 ± 0.34	0.33 ± 0.09	2.19 ± 0.43
	SKBR3	0.91 ± 0.03	1.10 ± 0.61	5.90 ± 2.13
Ovarian cancer	A2780	0.45 ± 0.22	0.60 ± 0.05	0.45 ± 0.05
	SKOV3	1.20 ± 0.90	1.03 ± 1.25	1.49 ± 0.93

Cancer cell lines were incubated in various concentrations of ponatinib, JQ1 or dBET1 at 37° C for 48 hours. Then, ^3^H-thymidine was added and the incorporated radioactivity was determined 16 hours later. Dose-dependent growth inhibition was seen in all cells. The table provides the IC_50_ values obtained with ponatinib, JQ1 and dBET1. Mean ± SD of 3 independent experiments are given.

### Drug effects on cell cycle progression in cancer cell lines

**Figure 1:**
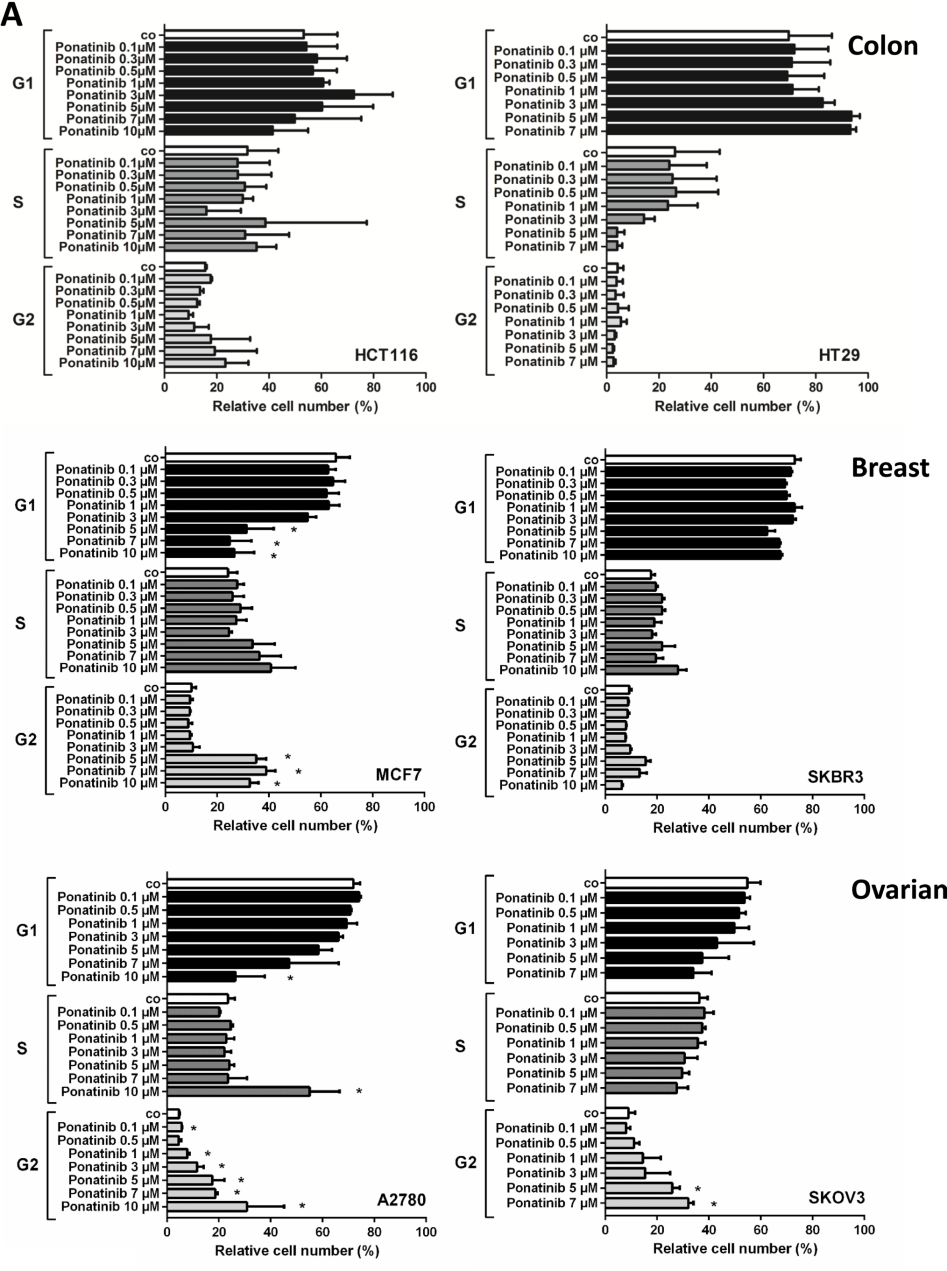

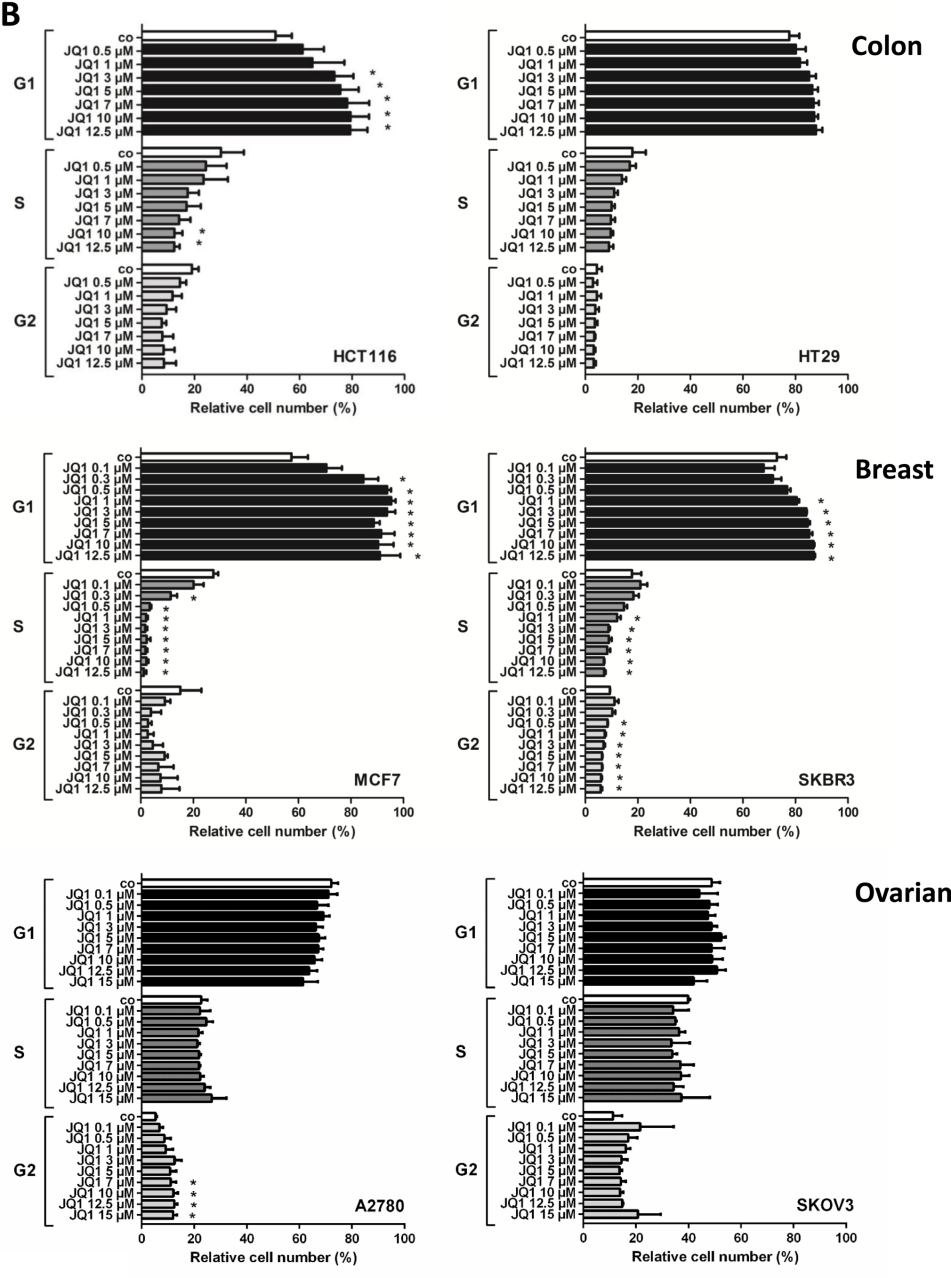

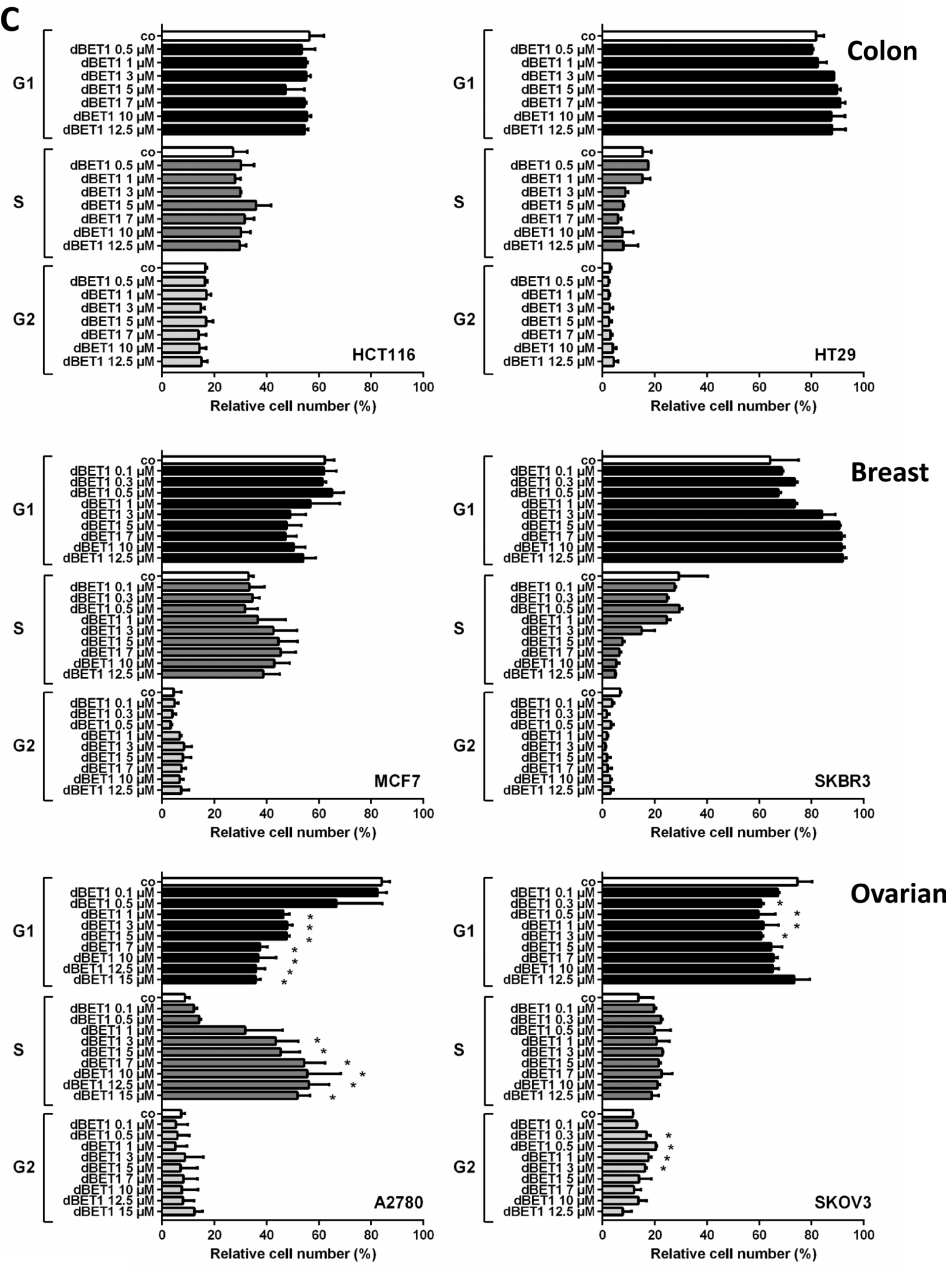
Effect of ponatinib, JQ1 and dBET1 on cell cycle distribution in colon, breast and ovarian cancer cells HCT116, HT29, MCF7, SKBR3, A2780 and SKOV3 cells were incubated in control medium (co) or in medium containing various concentrations of ponatinib (**A**), JQ1 (**B**) or dBET1 (**C**) at 37° C for 48 hours. Thereafter, cell cycle distribution was analyzed by flow cytometry. Results show relative cell numbers and represent the mean ± SD of 3 independent experiments. The level of significance was determined by ANOVA followed by Scheffe test. Asterisk (^*^): *p* < 0.05 compared to control.

To further examine the mechanisms of drug-induced growth arrest, cell-cycle progression was determined by flow cytometry using PI for DNA labeling. Ponatinib induced a dose-dependent G2/M arrest in ovarian and MCF7 breast cancer cells, but was not able to block cell cycle in colon and SKBR3 breast cancer cells (Figure 1A). JQ1, on the other hand, dose-dependently blocked the cell cycle at G0/G1 and reduced S and G2/M in colon and breast cancer cells, whereas in ovarian cancer cells the effects were less pronounced (Figure 1B). Similar effects on the cell cycle were observed with the BRD4 degrader dBET1 (Figure 1C).

### Effects of ponatinib and BET-targeting drugs on survival of cancer cells

**Figure 2:**
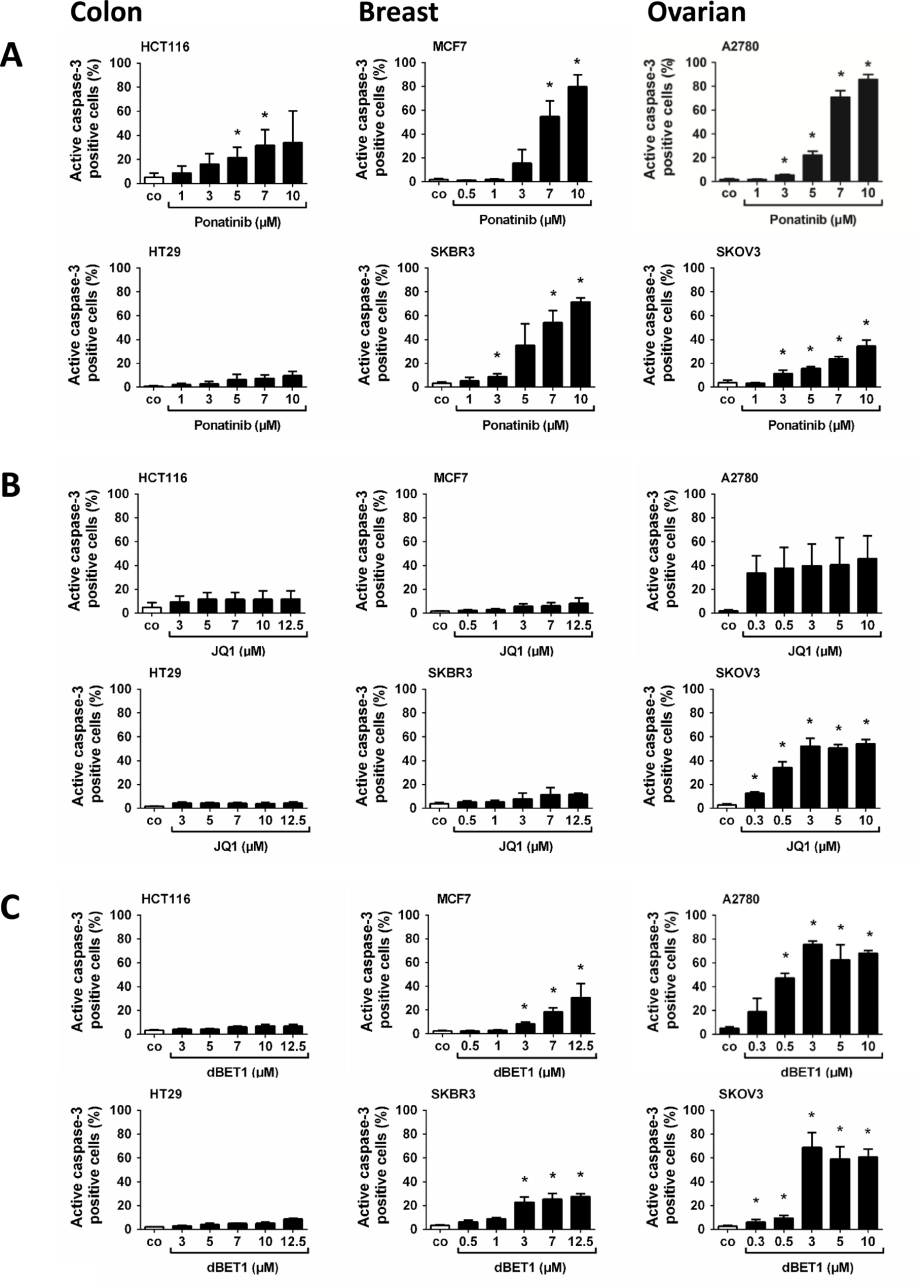
Effects of ponatinib, JQ1 and dBET1 on late apoptosis of colon, breast and ovarian cancer cells HCT116, HT29, MCF7, SKBR3, A2780 and SKOV3 cells were incubated in control medium (co) or in medium containing various concentrations of ponatinib (**A**), JQ1 (**B**) or dBET1 (**C**) at 37° C for 48 hours. Then, cells were examined by flow cytometry to determine the percentage of late apoptotic, active caspase-3 positive cells. Results represent the mean ± SD of 3 independent experiments. The level of significance was determined by ANOVA followed by Scheffe test. Asterisk (^*^): *p* < 0.05 compared to control.

In a next step, we examined whether the growth-inhibitory effects of ponatinib, JQ1 and dBET1 are associated with apoptosis. Drug-induced early and late apoptosis was quantified by flow cytometry of Annexin V- and active caspase-3-labelled cells, respectively. Although both data sets do not always match exactly, we can still draw some general conclusions. Ponatinib induced marked dose-dependent apoptosis in all cell lines tested except HT29 (Figure 2A and Supplementary Figure 1A). The BRD4 inhibitor JQ1 was a poor inducer of apoptosis (Figure 2B and Supplementary Figure 1B), whereas the BRD4 degrader dBET1 elicited mild, dose-dependent apoptosis in all cell lines (Figure 2C and Supplementary Figure 1C). For instance, the proportion of late apoptotic (active caspase-3-positive) A2780 cells amounted to 47,40 ± 3,06 % after treatment with 0.5 µM dBET1 relative to 5,12 ± 0,96 % in controls (Figure 2C) and the fraction of early apoptotic (Annexin V-positive) A2780 cells was 35,89 ± 1,21 % compared to 4,93 ± 1,23 % in controls, respectively (Supplementary Figure 1C). Generally, colon cancer cell lines appeared to be relatively insensitive to apoptosis induction by BRD4-targeting drugs, which corroborates recent data [10, 19].

### Drug-mediated anti-neoplastic effects are associated with inhibition of crucial upstream regulators and downstream effectors of carcinoma development and progression

**Figure 3:**
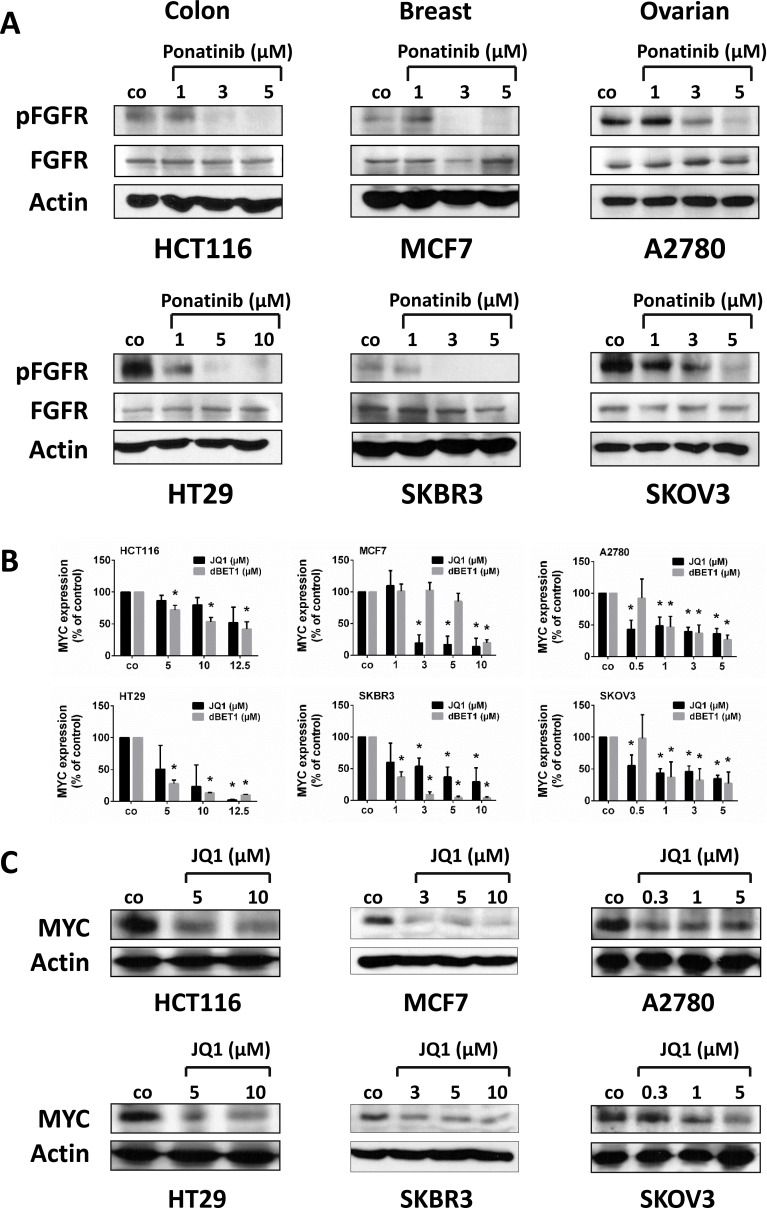
Effect of ponatinib on (p)FGFR expression and of JQ1 on MYC expression in colon, breast and ovarian cancer cells HCT116, HT29, MCF7, SKBR3, A2780 and SKOV3 cells were incubated in control medium (co) or in medium containing various concentrations of ponatinib (**A**), JQ1 (**B**, **C**) or dBET1 (B) at 37° C for 4 hours (A), 16 hours (B) or 24 hours (C), respectively. (A, C) Cells were harvested and examined for expression of pFGFR, FGFR and MYC by Western blotting using a polyclonal anti-phospho-FGFR antibody (Tyr653/654) (1:1000), a polyclonal anti-FGFR (1:1000) (A), or a monoclonal antibody against MYC (1:1000) (C). Equal loading was confirmed by using a polyclonal antibody against beta-actin (1:1000). pFGFR, phospho-FGFR. (B) Expression of *MYC* mRNA was determined by qRT-PCR analysis. The relative expression levels of *MYC* mRNA were calculated by the standard curve method and *Abl1* was used as internal control. The figures show the mean ± SD of 3 independent experiments. The level of significance was determined by ANOVA followed by Scheffe test. Asterisk (^*^): *p* < 0.05 compared to control.

Accumulating evidence suggests that ponatinib interferes with several oncogenic kinase targets, including members of the FGFR family. The FGF-FGFR growth and survival system is one of the key oncogenic signaling pathways in solid tumors and is known to be hyperactive in colon, breast and ovarian cancer [20]. Therefore, we examined the phosphorylation status of FGFR upon exposure of cancer cells to ponatinib. Indeed, ponatinib was found to abolish phosphorylation of FGFR in all tested cell lines in our Western blot analyses (Figure 3A), which correlates with induction of apoptosis in all cell lines except HT29 (Figure 2A and Supplementary Figure 1A).

JQ1, on the other hand, is a potent and well-known inhibitor of BRD4 that causes downregulation of MYC expression in various cell types [21], and dBET1 induces degradation of BRD4, which also leads to reduction of MYC expression [8]. In line with these observations, the anti-tumor effects of JQ1 and dBET1 were associated with a striking decrease in *MYC* mRNA- and MYC protein expression in all cell lines tested (Figure 3B and 3C).

### Combination of ponatinib with BRD4-targeting drugs potentiates growth inhibition and apoptosis in most carcinoma cell lines

**Figure 4:**
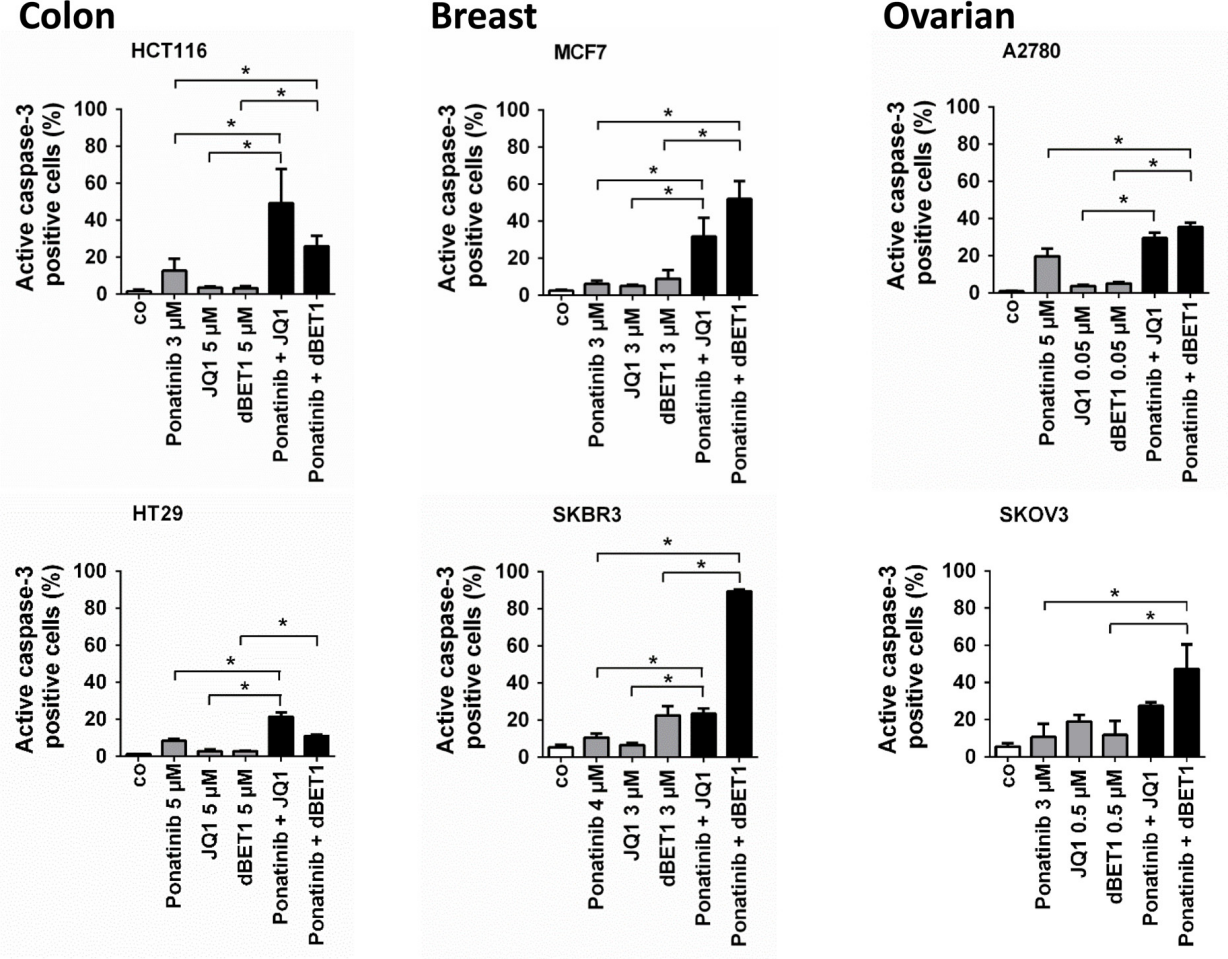
Effects of combination of ponatinib and JQ1 or ponatinib and dBET1 on late apoptosis of colon, breast and ovarian cancer cells HCT116, HT29, MCF7, SKBR3, A2780 and SKOV3 cells were incubated in control medium (co) or in medium containing ponatinib, JQ1, dBET1 or a combination of ponatinib + JQ1 or ponatinib + dBET1 at 37° C for 48 hours. Drug concentrations (EC_max/2_) causing approximately half maximum induction of apoptosis (E_max/2_) when given alone were chosen according to dose response-relationships given in Figure 2. For detailed information on the procedure for finding proper concentrations please see Supplementary Table 1. Cells were examined by flow cytometry to determine the percentage of late apoptotic, active caspase-3 positive cells. Results represent the mean ± SD of 3 independent experiments. The level of significance was determined by ANOVA followed by Scheffe test. Asterisk (^*^): *p* < 0.05.

In an attempt to augment the poor pro-apoptotic effects of the BRD4-targeting drugs, we combined JQ1 or dBET1 with the multi-TKI ponatinib. In each cell line, a drug concentration below or around the EC_max/2_ was chosen for examining drug-induced apoptosis in combination experiments. These doses caused half maximum apoptotic response in single treatments in the respective cell lines (E_max/2_) (Supplementary Table 1). Figure 4 and Supplementary Figure 2 show that ponatinib, when combined with JQ1 or dBET1, induced significantly more apoptosis than each drug alone in 5 of the 6 cell lines tested. In a next step, drug combination experiments applying a range of drug concentrations combined at fixed dose ratios, were performed. CompuSyn software was used to calculate combination indexes (CIs) in these experiments. The resulting CI values were close to or indicative of additive drug interactions. For instance, when ponatinib was combined with JQ1, Annexin V labelling revealed mostly additive effects in HCT116 cells (CI: 0.90–1.01) and MCF7 cells (CI: 0.91–0.98), while synergism, characterized by a CI value below 1, was only observed in SKOV3 cells (CI: 0.43–0.45). In HT29 cells, combined treatment resulted in an additive decrease in ^3^H-thymidine uptake and thus proliferation (CI: 0.90–1.22) but not in an additive decrease in cell survival (Supplementary Figure 3).

### Increased anti-cancer efficacy produced by combined application of ponatinib and BRD4-targeted drugs is associated with MYC hypersuppression in cancer cells

**Figure 5:**
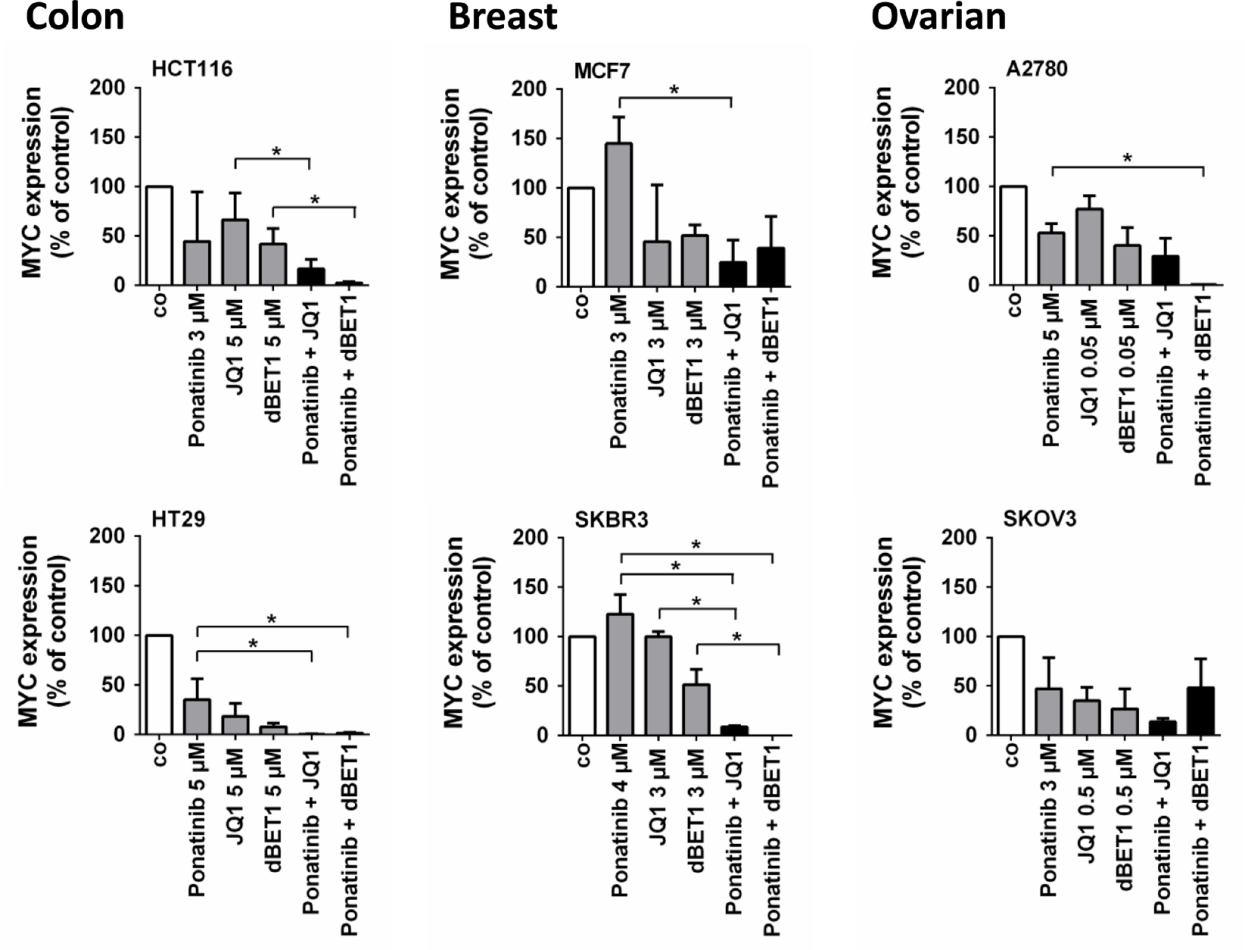
Effects of combination of ponatinib and JQ1 or ponatinib and dBET1 on MYC expression in colon, breast and ovarian cancer cells HCT116, HT29, MCF7, SKBR3, A2780 and SKOV3 cells were incubated in control medium (co) or in medium containing ponatinib, JQ1, dBET1 or a combination of ponatinib + JQ1 or ponatinib + dBET1 at 37° C for 16 hours. Drug concentrations (EC_max/2_) causing approximately half maximum induction of apoptosis (E_max/2_) when given alone were chosen according to dose response-relationships given in Figure 2. For detailed information on the procedure for finding proper concentrations please see Supplementary Table 1. *MYC* expression was determined by qRT-PCR analysis. The relative *MYC* expression levels were calculated by the standard curve method and *Abl1* was used as internal control. The figures show the mean ± SD of 3 independent experiments. The level of significance was determined by ANOVA followed by Scheffe test. Asterisk (^*^): *p* < 0.05.

We then asked whether the observed elevated anti-cancer efficacy after co-exposure to ponatinib and JQ1 or dBET1 is associated with stronger suppression of *MYC* oncogene expression. Indeed, the combination of drugs induced a significantly stronger downregulation of MYC than the individual drugs in most cancer cell lines tested, and in some cell lines, *MYC* was virtually eliminated after co-exposure (Figure 5).

## DISCUSSION

**Figure 6:**
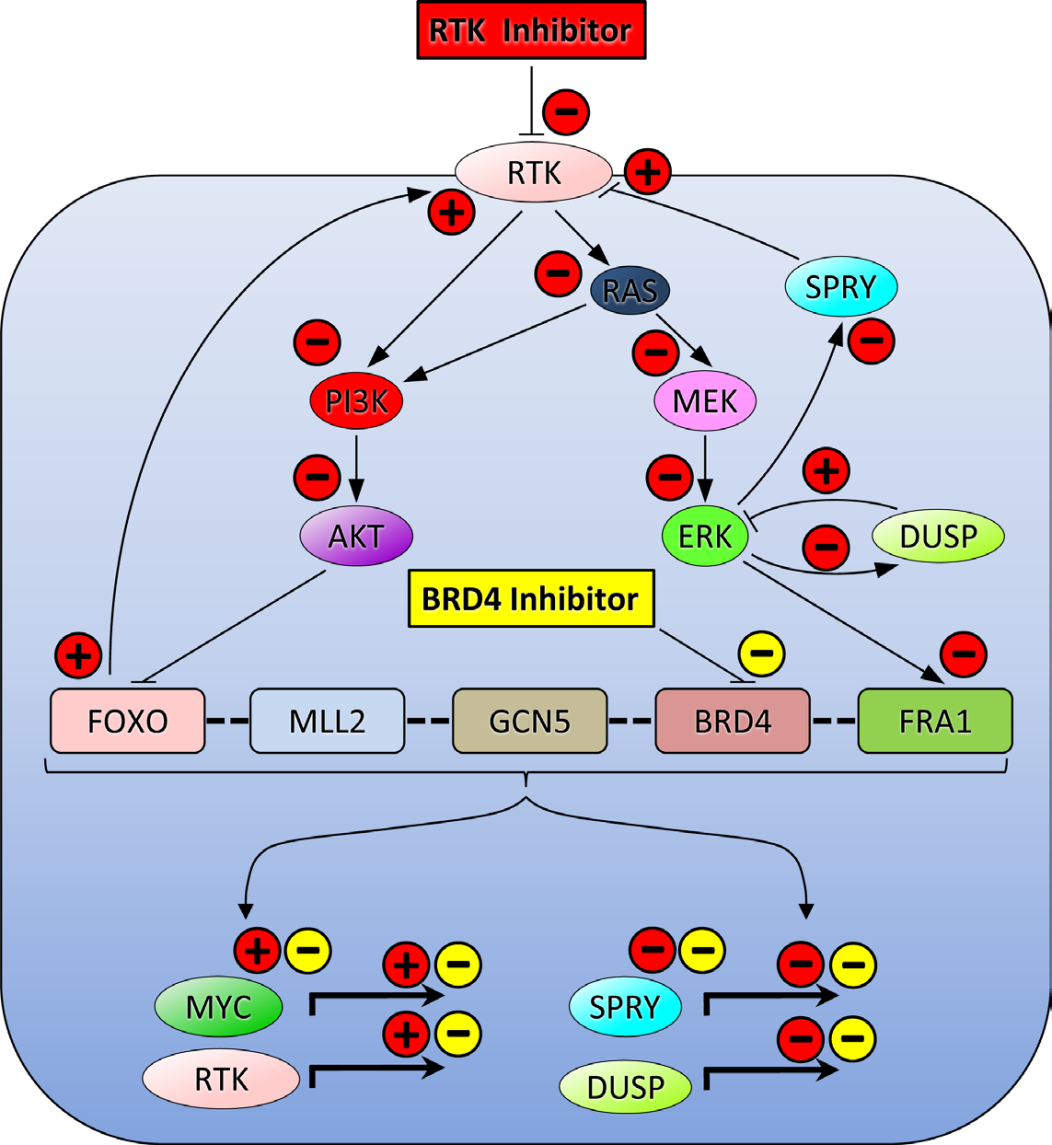
A model summarizing the mechanisms of cooperation between RTK signaling and BRD4-controlled epigenetic regulation of oncogene expression and activation reveals nodes for combined targeting in order to disable compensatory bypass routes RTKs are upstream activators of PI3K/AKT and MEK/ERK. AKT is known to block the interaction of FOXO transcription factors with the histone methyltransferase MLL2, the histone acetyltransferase GCN5 and the chromatin reader BRD4. The MAPK ERK, on the other hand, activates the terminal transcription factor FRA1, which–just like BRD4–binds to the MYC super-enhancer. Thus, all these transcription regulators cooperate with BRD4 in order to control the expression of oncogenes such as *MYC* and *RTKs*. Consequently, an RTK inhibitor (red rectangle), e.g. ponatinib, abrogates PI3K/AKT and MEK/ERK/FRA1 (red minus signs) neutralizing FOXO-, SPRY-, and DUSP-mediated negative feedback regulation of RTK and ERK and enabling transcription of *MYC* and *RTKs* (red plus signs), while abrogating the transcription of *SPRY* and *DUSP*, two negative modulators of RTK and ERK, respectively (red minus signs). Importantly, these feedback loops can be impeded by concurrent exposure to BRD4 inhibitors such as JQ1 or dBET1 (yellow rectangle), which diminish transcription of *MYC* and *RTKs*, and of RTK- and ERK-antagonistic genes such as *SPRY* and *DUSP* (yellow minus signs). Pointed or blunt arrows indicate stimulation or inhibition, respectively. Bold dashed lines signify assembly of the transcriptional regulator complex (bracket). Bold right-angle arrows depict gene transcription. Abbreviations: BRD4, bromodomain-containing protein 4; DUSP, dual specificity protein phosphatase; ERK, extracellular signal-regulated kinase; FOXO, forkhead box protein O; FRA1, fos-related antigen 1; GCN5, general control of amino acid synthesis protein 5; MAPK, mitogen-activated protein kinase; MEK, dual specificity mitogen-activated protein kinase kinase; MLL2, mixed-lineage leukemia protein 2; MYC, myelocytomatosis oncogene; PI3K, phosphatidylinositol-4,5-bisphosphate 3-kinase; RAS, rat sarcoma oncogene; RTK, receptor tyrosine kinase; SPRY, sprouty.

Oncogenic signal transduction from transmembrane receptors along cytoplasmic PI3K-AKT-mTORC1 and RAF-MEK-MAPK serine/threonine kinase cascades down to transcription factors such as MYC is a critical signaling system in many carcinomas. This highlights the central role of PI3K and MAPK in the development and progression of solid tumors. Hence, blockade of these pathways has been considered a prime goal for improving cancer treatment. Unfortunately, however, most cancer cells rapidly develop resistance to kinase inhibitors, due, at least in part, to drug-mediated disruption of negative feedback loops toward activating upstream receptors. Thereby, signal transduction can be restored despite concurrent blockade of downstream kinases [22–25]. Such types of compensatory bypass routing and rewiring of the kinome causing treatment failure may also occur when solid tumors are exposed to BET inhibitors [26]. Remarkably, when EGFR/ERBB2 inhibitors, PI3K blockers or MAPK antagonist drugs were co-administered with BET inhibitors, antineoplastic effects were stronger compared to exposure to individual drugs [10, 12, 19, 27–29]. This drug interaction could be explained by a variety of underlying mechanisms (Figure 6). A pharmacological blockade of PI3K alleviates BET-driven feedback inhibition of receptor tyrosine kinases (RTKs) and stimulates the FOXO-BRD4-MYC axis. Abrogation of this negative feedback loop induces counteractive stimulation of RTKs and leads to upregulation of MYC [27, 29]. Alternatively, specific transcription factors representing terminal components of the MAPK pathway such as FRA1 bind to promoters of target genes including MYC that harbor super-enhancer sequences, which are particularly sensitive to BET inhibition [19]. Evidence suggests that RTK and BET pathways are linked *via* the transcription factors FOXO and FRA1, which regulate expression and function of both RTKs and MYC [29]. This observation led us to hypothesize that this type of paradox kinase inhibitor-dependent activation of RTKs and MYC could be eliminated by concomitant treatment with drugs that target BET family proteins such as BRD4. Therefore, we have used a combinatorial strategy that affects both signal generation at the membrane as well as downstream gene transcription in the nucleus. The consequences of these pharmacological interventions and the corresponding positive and negative interactions between the key players in this complex regulatory feedback system are summarized in Figure 6. Ponatinib binds and blocks multiple TKs directly. Its target affinity is highest for ABL, moderate for FGFR1, PDGFRα, VEGFR2, SRC and KIT, and lower for FLT3 [13, 14]. Ponatinib was therefore a perfect candidate for combination with BRD4 inhibitors such as JQ1 or dBET1. JQ1 targets MYC indirectly by displacing BRD4 from the acetylated chromatin at the *MYC* gene locus, while the novel BET degrader dBET1 induces highly selective cereblon-dependent BRD4 protein degradation, which also results in downregulation of MYC [8, 9]. Remarkably, until the availability of JQ1 and derivatives, MYC was considered a non-druggable cancer target. Although BET inhibitors only indirectly attack MYC, they are currently the most advanced MYC antagonizing drugs.

Ponatinib has been approved for use against TKI resistant Ph+ CML and Ph+ ALL [30] and due to its affinity for FGFR, which is regularly hyperactive in solid tumors, ponatinib can also induce moderate growth arrest and apoptosis in solid tumors such as neuroblastoma and endometrial cancer [31, 32]. We observed that ponatinib exerts anti-proliferative and pro-apoptotic activity in colon, breast and ovarian cancer. Ponatinib may thus be applicable for treatment of a wide variety of malignant diseases. Using Western blot analysis we demonstrate that treatment-naïve colon, breast and ovarian cancer cells typically reveal high levels of FGFR activity (phosphorylation). Interestingly, hyperactive FGFR signaling was abrogated by ponatinib, consistent with previous data from neuroblastoma and endometrial cancer [31, 32]. Dose-dependent downregulation of the activated (phosphorylated) form of FGFR correlated with induction of programmed cell death. This indicates that the FGFR function is crucial for the oncogenic growth of these cells and that FGFR may be a suitable therapeutic target in solid tumors. As stated above, the multi-TKI ponatinib co-inhibits additional (receptor) tyrosine kinases depending on distinct cell-type specific patterns of kinase signaling. Interestingly, the sensitivity of solid tumor cells against ponatinib was found to be markedly lower than that reported earlier for hematological cancer cells [33, 34]. This is probably due to the relatively high affinity of ponatinib for ABL, which is not normally hyperactive in solid tumors compared to all other targets of ponatinib [13].

Compounds that interfere with the transcription machinery at sites of activated chromatin represent another recently emerging group of promising anti-cancer drugs. JQ1, the prototype in this class of therapeutics, blocks interaction of the BET family protein BRD4 with acetylated histones and transcription factors. Our data demonstrate that JQ1-mediated growth inhibition is accompanied by G0/G1 cell cycle arrest and downregulation of MYC, whereas apoptosis is only weakly activated by JQ1. This is perfectly in line with previous findings in murine and human models of thyroid cancer, oral squamous cell carcinoma and mesenchymal stem cells [35–39].

The recently developed BET degraders are promising new tools for endogenous BET target protein degradation. dBET1, for example, represents a chimeric molecule generated by covalent linkage of JQ1 with thalidomide. The JQ1 part of the molecule interacts with BRD4, while the phthalimide moiety of the thalidomide part triggers subsequent degradation of BRD4 *via* activation of cereblon–a component of the ubiquitin ligase complex [40]. Winter *et al.* were the first to compare dBET1 with JQ1 in hematological malignancies. Compared to JQ1, they found that dBET1 induced much stronger apoptosis in AML and lymphoma cells and revealed improved *in vivo* antitumor efficacy in a human leukemia xenograft model [8]. Accordingly, we observed in the present study with cells from solid tumors that dBET1 outperformed JQ1 in induction of apoptosis (cf. Figure 2B with 2C and Supplementary Figure 1B with 1C).

In addition to overexpression of oncogenic transcription factors such as MYC, hyperactive pro-survival signaling *via* MAPK and PI3K contributes to the increased tumorigenicity of solid tumors [41, 42]. MYC is mainly activated by BRD4, whereas MAPK and PI3K are stimulated by membrane RTKs such as FGFR. Therefore, pharmacological targeting of upstream inducers such as BRD4 and FGFR is an efficient way to eliminate MYC, MAPK and PI3K function, respectively. Yet, reports of the combination of TKIs with BET inhibitors/degraders are still scarce, especially in solid tumors [10, 12, 19, 27, 43]. Here, we provide evidence that a combination of the TKI ponatinib with the BRD4 inhibitor JQ1 or the BRD4 degrader dBET1 significantly induces apoptosis in colon, breast and ovarian cancer cells. AKT, an important downstream effector of FGFR, promotes cell survival by activating the p53 repressor MDM2 and blocking the pro-apoptotic proteins BAD and BIM [44]. Ponatinib thus affects cancer cell growth by blocking the FGFR/AKT/MDM2 survival pathway, resulting in the activation of BIM-mediated apoptosis. MYC is overexpressed in a wide variety of human cancers. It stimulates growth and promotes cell cycle progression. Accordingly, our data demonstrate that JQ1- and dBET1-mediated MYC inhibition causes cell cycle arrest. Intriguingly, ponatinib and JQ1 or dBET1 appear to converge on growth and survival pathways, respectively, significantly enhancing apoptosis relative to single drug treatment. It seems therefore that impairment of kinase signaling aggravated anti-BRD4 mediated apoptosis in these cancer cell lines. This indicates that kinase transduction systems normally cooperate with BRD4 pathways to ensure survival of solid tumor cells. Notably, HT29 cells show a slightly different sensitivity. At low concentrations of ponatinib (≤1 µM) the growth of HT29 cells was blocked in the absence of noticeable apoptosis and cell cycle arrest. A similar phenomenon has been observed earlier, when growth arrest was triggered by inhibitors of metabolic enzymes [45–47]. According to these studies growth inhibition is caused by a generalized cell cycle delay that consistently prolongs the transit times through all phases of the cycle and may even lead to a complete stoppage of the cell cycle in the absence of accumulation of the cells in any specific phase of the cycle. In contrast, increasing the concentration of ponatinib beyond 1µM appears to induce G0/G1 cell cycle arrest in HT29 cells (Figure 1A). Another interesting phenomenon is that dBET1, which interferes with the chromatin reader protein BRD4, causes 1) growth arrest (Table 1), 2) accumulation of the cells in the S-phase of the cell cycle (Figure 1C), and 3) apoptosis in A2780 cells (Figure 2C and Supplementary Figure 1C). These results are in line with previous findings showing that chromatin- and/or DNA-targeting agents can decelerate/halt S phase transition [48, 49] followed by activation of apoptosis [49, 50]. Therefore we conclude that dBET1 inhibits A2780 cell growth by S phase cell cycle arrest followed by programmed cell death. Our results suggest broader use of these inhibitors not only for hematologic malignancies for which they have been developed, but also for solid tumors. Intriguingly, the data supports our hypothesis that the simultaneous targeting of two major oncogenic machineries is highly effective. Therefore, if both types of drugs were combined, lower drug concentrations were required to achieve the same antineoplastic response. Combination regimens would therefore produce less toxic side effects and would be less harmful to the patient and should be further examined in clinical trials for colorectal, breast and ovarian cancer as well as other malignancies.

## MATERIALS AND METHODS

### Reagents

The TKI ponatinib and the BRD4 inhibitor JQ1 were purchased from Selleckchem (Houston, TX). The potent and selective BRD4 degrader dBET1 was purchased from Chemietek (Indianapolis, IN). Dulbecco’s modified Eagle’s medium (DMEM), *α-modified* minimal essential medium (α-MEM) and phosphate buffered saline (PBS) were obtained from Gibco Life Technologies (Gaithersburg, MD). RPMI 1640 medium and fetal calf serum (FCS) were purchased from PAA Laboratories (Pasching, Austria). ^3^H-thymidine was from Amersham (Buckinghamshire, UK), Annexin V-FITC from eBiosciences (San Diego, CA) and the PE-labeled anti-active caspase-3 antibody C92-605 from BD Biosciences (San Jose, CA). Propidium iodide (PI) and RNase A were from Sigma (St. Louis, MO) and Triton X-100 from Promega (Mannheim, Germany). Monoclonal rabbit anti-MYC (D84C12), polyclonal rabbit anti-phospho-FGFR (Tyr653/654) and polyclonal rabbit anti-FGFR were purchased from Cell Signaling Technology (Danvers, MA), whereas polyclonal goat anti-actin and peroxidase-conjugated bovine anti-goat IgG were obtained from Santa Cruz Biotechnology (Dallas, TX), and peroxidase-conjugated donkey anti-rabbit IgG was from GE Healthcare (Buckinghamshire, UK).

### Cell lines and culture conditions

Colon (HCT116, HT29), breast (MCF7, SKBR3), and ovarian (A2780, SKOV3) cancer cell lines were cultured in DMEM (HCT116, MCF7, SKBR3), RPMI 1640 (HT29, A2780) or α-MEM (SKOV3) supplemented with 10% FCS and antibiotics at 37° C in 5% CO_2_. Cells were tested for absence of viral/bacterial/fungal/mycoplasma infection by Venor GeM (Minerva Biolabs, Berlin, Germany). The species origins were proven by species-PCR, and cell line identities were examined by fluorescent nonaplex-PCR of short tandem repeat markers (DSMZ, Braunschweig, Germany).

### Measurement of ^3^H-thymidine incorporation

To determine growth-modulating effects of ponatinib, JQ1 and dBET1, cell proliferation was analyzed by measuring ^3^H-thymidine uptake as described [51, 52]. Briefly, cell lines were exposed to ponatinib (0.001–10 µM) and/or JQ1 (0.05–10 µM) or dBET1 (0.05–10 µM) at 37° C for 48 hours. Thereafter, 1 µCi (0.037 MBq) ^3^H-thymidine was added (37° C, 16 hours). Then, cells were harvested on filter membranes (Packard Bioscience, Meriden, CT) in a Filtermate 196 harvester (Packard Bioscience). Filters were air-dried, and the bound radioactivity was counted in a β-counter (Top-Count NXT, Packard Bioscience). All experiments were performed in triplicates.

### Apoptosis assays

For flow cytometric determination of apoptosis, Annexin V staining and active caspase-3 labeling was performed. Cell lines were incubated in medium without or with ponatinib (0.5–10 µM), JQ1 (0.3–12.5 µM), dBET1 (0.3–12.5 µM) or a combination of ponatinib + JQ1 or ponatinib + dBET1 at 37° C for 48 hours. For detection of early apoptosis drug-induced externalization of phosphatidylserine was determined. To this end, cells were harvested after drug exposure, washed with PBS and incubated with Annexin V-FITC in binding-buffer containing HEPES (10 mM, pH 7.4), NaCl (140 mM) and CaCl_2_ (2.5 mM). Then, cells were washed and PI (1 mg/mL) was added to exclude necrotic material from analysis. Cells were examined by flow cytometry on a FACSCalibur (BD Biosciences). To quantify active caspase-3-positive (late apoptotic) cells, cultures were fixed in formaldehyde (2%) and permeabilized using methanol (100%) for 15 minutes at –20° C. After staining with PE-labeled anti-active caspase-3, cells were analyzed by flow cytometry on a FACSCalibur.

### Cell cycle analyses

Cells were incubated in medium without or with ponatinib (0.1–10 µM), JQ1 (0.1–12.5 µM) or dBET1 (0.1–12.5 µM) at 37° C for 48 hours. Thereafter, cells were harvested, washed in PBS and fixed in 70% ethanol for 15 minutes at 4° C. Fixed cells were washed and incubated in 0.05% Triton X-100/PBS, RNase A (100 μg/mL) and PI (50 µg/mL) for 40 minutes at 37° C. Then, cells were washed with PBS and analyzed using a FACSCalibur. Data were analyzed using ModFit software (Verity Software House, Topsham, ME).

### Quantitative reverse transcription-PCR (qRT-PCR)

Cell lines were incubated with various concentrations of ponatinib, JQ1, dBET1 or a combination of ponatinib + JQ1 or ponatinib + dBET1 at 37° C for 16 hours. Total RNA was isolated using the RNeasy Mini Kit (Qiagen, Hilden, Germany) according to the manufacturer’s protocol. Reverse transcription was performed using Moloney murine leukemia virus reverse transcriptase, random primers, first strand buffer (Invitrogen, Carlsbad, CA), dNTPs (Promega, Mannheim, Germany) and RNasin plus (Promega) according to manufacturer’s instructions (Invitrogen). qRT-PCR was performed using primers (Eurofins MWG Operon, Ebersberg, Germany) for human *ABL1* (forward: 5′-TGTATGATTTTGTGGCCAGTGGAG-3′; reverse: 5′-GCCTAAGACCCGGAGCTTTTCA-3′) and human *MYC* (forward: 5′-TGCTCCATGAGGAGACACC-3′; reverse: 5′-CCTGCCTCTTTTCCACAGAA-3′). mRNA levels were quantified on a 7900HT Fast Real-Time PCR System (Applied Biosystems, Foster City, CA) using iTAq SYBR Green Supermix with ROX (Bio-Rad, Hercules, CA). Expression levels of *MYC* were normalized to *ABL1* by the standard curve method [53].

### Western blot analyses

Cells were incubated with ponatinib (1–10 µM) at 37° C for 4 hours or with JQ1 (0.3–10 µM) at 37° C for 24 hours. Western blotting was performed as described previously [51] using monoclonal rabbit anti-MYC (D84C12) (1:1000), polyclonal rabbit anti-phospho-FGFR (Tyr653/654) (1:1000) or polyclonal rabbit anti-FGFR (1:1000). Equal loading was verified with polyclonal goat anti-actin (1:1000). Antibody binding was visualized by peroxidase-conjugated donkey anti-rabbit IgG (1:10000) or peroxidase-conjugated bovine anti-goat IgG (1:10000) and enhanced chemiluminescence.

### Statistic and data analysis

Data are expressed as mean values with standard deviation from at least 3 independent experiments. Statistical analysis was performed by using ANOVA and post hoc Scheffe test.

The CompuSyn software (ComboSyn, Paramus, NJ) is based on the classical median effect analysis and the isobologram algorithm of Chou, Talalay and Sacks [54–56]. This mathematical method provides a combination index (CI) that characterizes the type of drug interaction in combination experiments when both drugs are used in different concentrations but in a fixed ratio. A CI value below 1, equal to 1 or above 1, means synergism, additivity or antagonism.

## SUPPLEMENTARY MATERIALS FIGURES AND TABLE


