# Supplementary material for: Hitting two oncogenic machineries in cancer cells: cooperative effects of the multi-kinase inhibitor ponatinib and the BET bromodomain blockers JQ1 or dBET1 on human carcinoma cells
Source: Oncotarget. 2018 May 29;9(41):26491–506. doi: 10.18632/oncotarget.25474 (PMC5995173; doi:10.18632/oncotarget.25474)
Supplement: Supplementary file 1 [file oncotarget-09-26491-s001.pdf]

# Hitting two oncogenic machineries in cancer cells: cooperative effects of the multi-kinase inhibitor ponatinib and the BET bromodomain blockers JQ1 or dBET1 on human carcinoma cells

## SUPPLEMENTARY MATERIALS

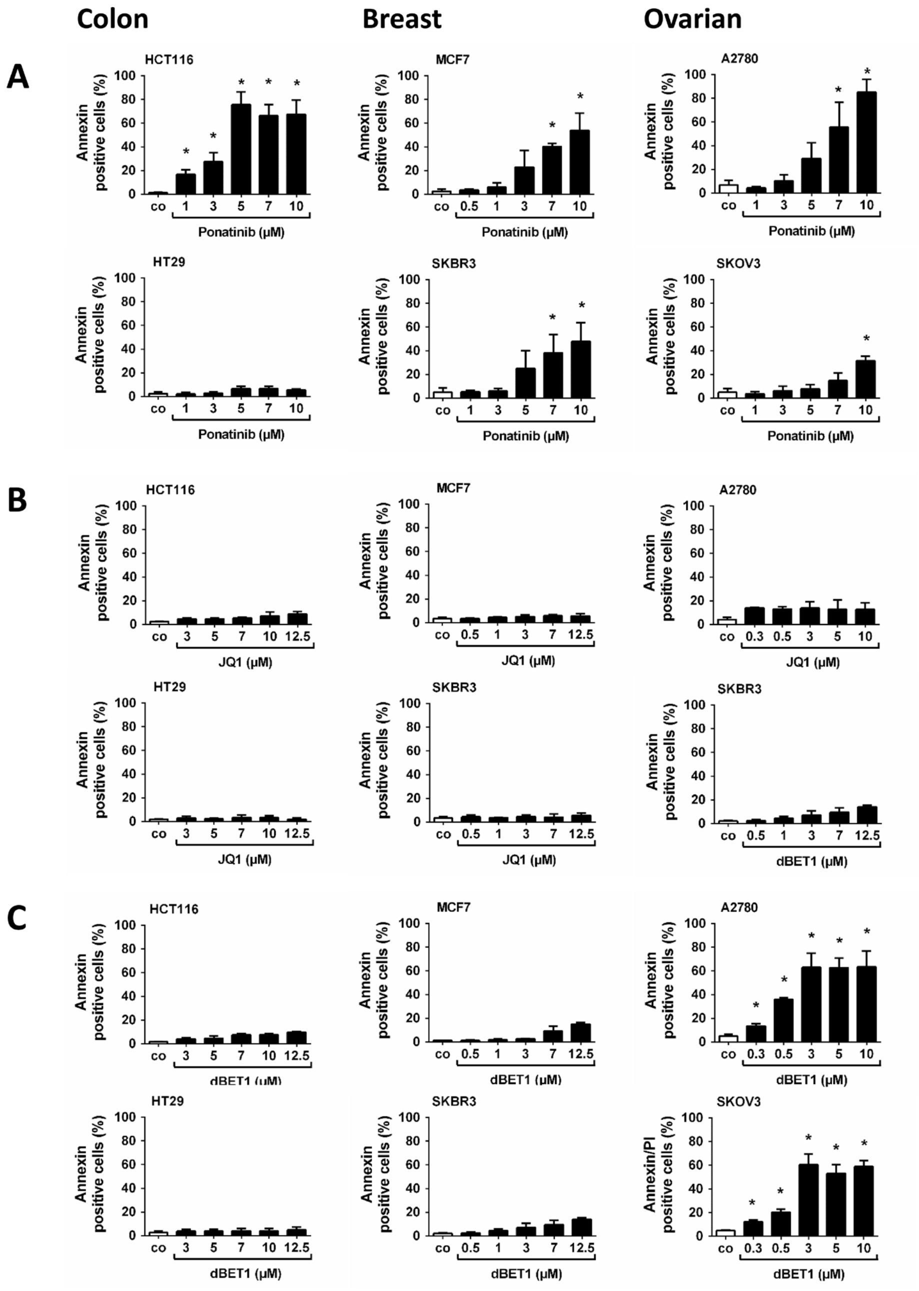


**Supplementary Figure 1: Effects of ponatinib, JQ1 and dBET1 on early apoptosis of colon, breast and ovarian cancer cells.** HCT116, HT29, MCF7, SKBR3, A2780 and SKOV3 cells were incubated in control medium (co) or in medium containing various concentrations of ponatinib (**A**), JQ1 (**B**) or dBET1 (**C**) at 37° C for 48 hours. Then, cells were examined by flow cytometry to determine the percentage of early apoptotic, Annexin V-positive cells. Results represent the mean ± SD of 3 independent experiments. The level of significance was determined by ANOVA followed by Scheffe test. Asterisk (*): $p < 0.05$ compared to control.

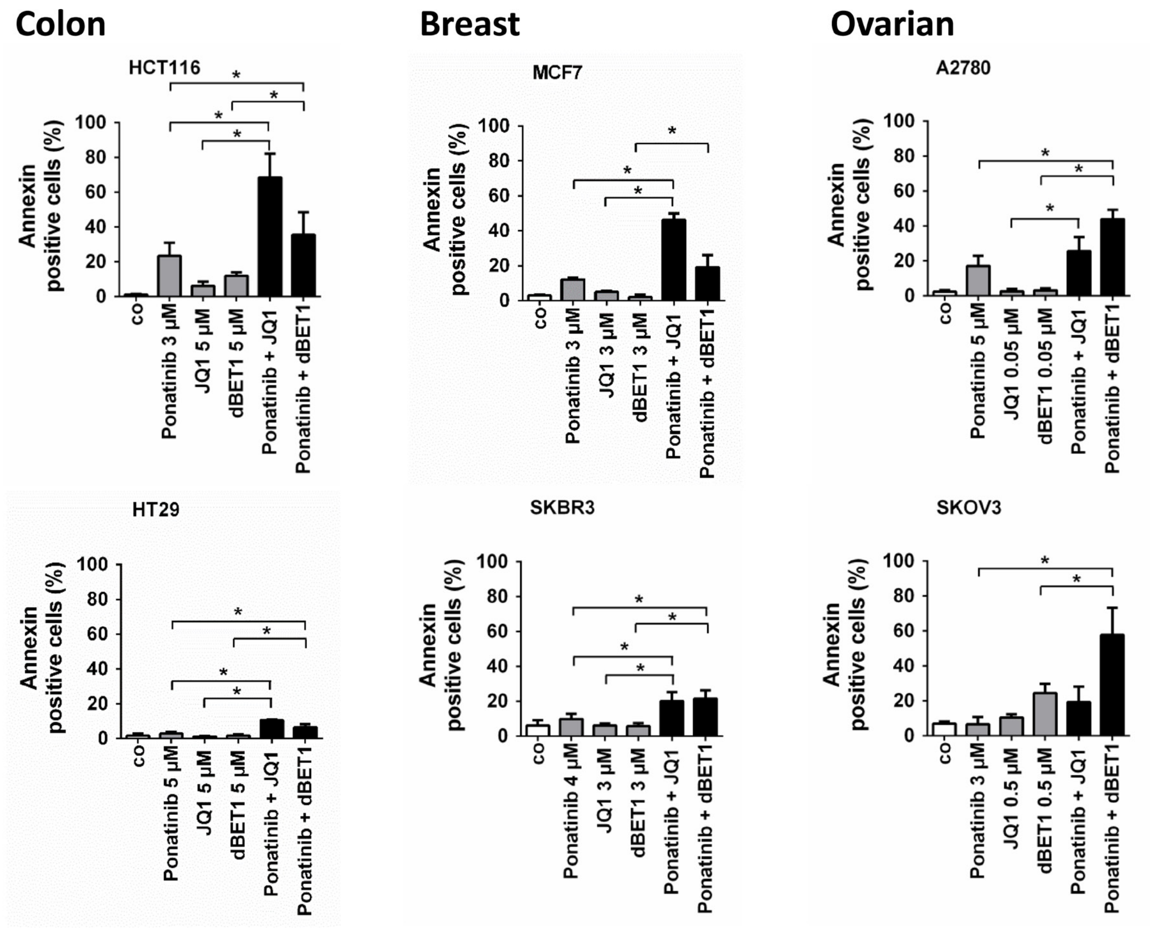


**Supplementary Figure 2: Effects of combination of ponatinib and JQ1 or ponatinib and dBET1 on early apoptosis of colon, breast and ovarian cancer cells.** HCT116, HT29, MCF7, SKBR3, A2780 and SKOV3 cells were incubated in control medium (co) or in medium containing ponatinib, JQ1, dBET1 or a combination of ponatinib + JQ1 or ponatinib + dBET1 at 37° C for 48 hours. Drug concentrations ($EC_{max/2}$) causing approximately half maximum induction of apoptosis ($E_{max/2}$) when given alone were chosen according to dose response-relationships given in Figure 2. For detailed information on the procedure for finding proper concentrations please see Supplementary Table 1. Cells were examined by flow cytometry to determine the percentage of early apoptotic, Annexin V-positive cells. Results represent the mean ± SD of 3 independent experiments. The level of significance was determined by ANOVA followed by Scheffe test. Asterisk ($^*$): $p < 0.05$.

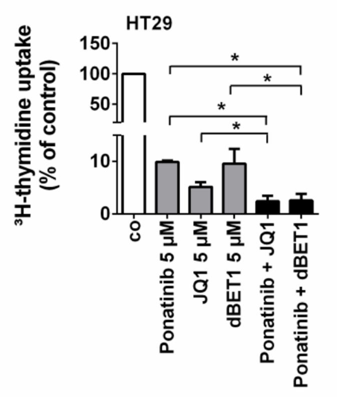


**Supplementary Figure 3: Effects of combination of ponatinib and JQ1 or ponatinib and dBET1 on proliferation of HT29 colon cancer cells.** HT29 cells were incubated in control medium (co) or in medium containing ponatinib, JQ1, dBET1 or a combination of ponatinib + JQ1 or ponatinib + dBET1 at 37° C for 48 hours. Drug concentrations ($EC_{max/2}$) causing approximately half maximum induction of apoptosis ($E_{max/2}$) when given alone were chosen according to dose response-relationships given in Figure 2. For detailed information on the procedure for finding proper concentrations please see Supplementary Table 1. Thereafter, $^3$H-thymidine uptake was measured. Results are expressed as percent of control (co) and represent the mean ± SD of 3 independent experiments. The level of significance was determined by ANOVA followed by Scheffe test. Asterisk ($^*$): $p < 0.05$.

**Supplementary Table 1: Determination of proper dosing of individual drugs in the combination experiments (table columns with headings 'Used Dose')**

**Active Caspase 3 Assay**

| | Ponatinib | | | | JQ1 | | | | dBET1 | | | |
|---|---|---|---|---|---|---|---|---|---|---|---|---|
| **Cell Line** | **Emax (%)** | **Emax/2 (%)** | **ECmax/2 (µM)** | **Used Dose (µM)** | **Emax (%)** | **Emax/2 (%)** | **ECmax/2 (µM)** | **Used Dose (µM)** | **Emax (%)** | **Emax/2 (%)** | **ECmax/2 (µM)** | **Used Dose (µM)** |
| **HCT116** | 33,80 | 16,90 | 3,50 | 3,00 | 11,80 | 5,90 | 3,40 | 5,00 | 6,60 | 3,30 | 4,60 | 5,00 |
| **HT29** | 9,60 | 4,80 | 3,50 | 5,00 | 4,20 | 2,10 | 3,70 | 5,00 | 8,60 | 4,30 | 5,60 | 5,00 |
| **MCF7** | 79,60 | 39,80 | 7,20 | 3,00 | 8,00 | 4,00 | 3,30 | 3,00 | 30,40 | 15,20 | 5,80 | 3,00 |
| **SKBR3** | 71,60 | 35,80 | 6,60 | 4,00 | 11,60 | 5,80 | 2,80 | 3,00 | 27,20 | 13,60 | 2,40 | 3,00 |
| **A2780** | 85,60 | 42,80 | 7,20 | 5,00 | 45,60 | 22,80 | 0,05 | 0,05 | 68,00 | 34,00 | 0,32 | 0,05 |
| **SKOV3** | 34,20 | 17,10 | 6,10 | 3,00 | 54,00 | 27,00 | 0,47 | 0,50 | 68,80 | 34,40 | 1,20 | 0,50 |

**Annexin V Assay**

| | Ponatinib | | | | JQ1 | | | | dBET1 | | | |
|---|---|---|---|---|---|---|---|---|---|---|---|---|
| **Cell Line** | **Emax (%)** | **Emax/2 (%)** | **ECmax/2 (µM)** | **Used Dose (µM)** | **Emax (%)** | **Emax/2 (%)** | **ECmax/2 (µM)** | **Used Dose (µM)** | **Emax (%)** | **Emax/2 (%)** | **ECmax/2 (µM)** | **Used Dose (µM)** |
| **HCT116** | 67,40 | 33,70 | 3,40 | 3,00 | 8,60 | 4,30 | 4,60 | 5,00 | 9,60 | 4,80 | 5,90 | 5,00 |
| **HT29** | 5,40 | 2,70 | 4,30 | 5,00 | 1,80 | 0,90 | 3,10 | 5,00 | 4,60 | 2,30 | 3,20 | 5,00 |
| **MCF7** | 53,80 | 26,90 | 6,30 | 3,00 | 5,60 | 2,80 | 2,70 | 3,00 | 15,00 | 7,50 | 4,20 | 3,00 |
| **SKBR3** | 47,60 | 23,80 | 6,20 | 4,00 | 5,40 | 2,70 | 2,10 | 3,00 | 14,00 | 7,00 | 6,00 | 3,00 |
| **A2780** | 85,00 | 42,50 | 6,60 | 5,00 | 12,40 | 6,20 | 0,03 | 0,05 | 63,20 | 31,60 | 0,44 | 0,05 |
| **SKOV3** | 31,40 | 15,70 | 7,00 | 3,00 | 22,40 | 11,20 | 0,45 | 0,50 | 58,80 | 29,40 | 0,72 | 0,50 |

The following procedure was chosen. First, in dose-response experiments spanning ranges from 0–10 or 0–12.5 µM (higher concentrations are not useful from a pharmacological point of view) we determined the maximally achievable apoptotic response ($E_{max}$) in % positive cells for each drug in each cell line. This value varied considerably between cell lines and compounds. Then, depending on the actual shape of the dose-response curves we applied linear or nonlinear regression analysis using the LOGEST or the LINEST functions in MS Excel in order to find best curve fitting functions and to calculate the drug concentrations ($EC_{max/2}$) in µM that elicit half-maximum apoptotic response ($E_{max/2}$) in % positive cells. Based on these results we have chosen single drug doses in µM (table columns with headings 'Used Dose') that satisfy the following criteria:

1. Used doses should be roughly around or below $EC_{max/2}$.
2. Used doses should be equal for both active caspase 3 and annexin V assays.
3. Used doses should be equal for both JQ1 and dBET1.

This procedure was used for obtaining data presented in Figures 4 and 5, and in Supplementary Figures 2 and 3.
